# Emerging Evidence of Noncoding RNAs in Bleb Scarring after Glaucoma Filtration Surgery

**DOI:** 10.3390/cells11081301

**Published:** 2022-04-12

**Authors:** Sabrina Yu, Alex L. C. Tam, Robert Campbell, Neil Renwick

**Affiliations:** 1 Faculty of Medicine, University of British Columbia, Vancouver, BC V6T 1Z4, Canada; sabrina.yu@alumni.ubc.ca; 2 Department of Ophthalmology and Visual Sciences, Queen’s University, Kingston, ON K7L 3N6, Canada; alex.tam@queensu.ca (A.L.C.T.); rob.campbell@queensu.ca (R.C.); 3 Department of Pathology and Molecular Medicine, Queen’s University, Kingston, ON K7L 3N6, Canada

**Keywords:** glaucoma, fibrosis, microRNAs, human tenon fibroblasts

## Abstract

Purpose: To conduct a narrative review of research articles on the potential anti- and pro-fibrotic mechanisms of noncoding RNAs following glaucoma filtration surgery. Methods: Keyword searches of PubMed, and Medline databases were conducted for articles discussing post-glaucoma filtration surgeries and noncoding RNA. Additional manual searches of reference lists of primary articles were performed. Results: Fifteen primary research articles were identified. Four of the included papers used microarrays and qRT-PCR to identify up- or down-regulated microRNA (miRNA, miR) profiles and direct further study, with the remainder focusing on miRNAs or long noncoding RNAs (lncRNAs) based on previous work in other organs or disease processes. The results of the reviewed papers identified miR-26a, -29b, -139, -155, and -200a as having anti-fibrotic effects. In contrast, miRs-200b and -216b may play pro-fibrotic roles in filtration surgery fibrosis. lncRNAs including H19, NR003923, and 00028 have demonstrated pro-fibrotic effects. Conclusions: Noncoding RNAs including miRNAs and lncRNAs are emerging and promising therapeutic targets in the prevention of post-glaucoma filtration surgery fibrosis.

## 1. Introduction

Glaucoma is a leading cause of irreversible blindness. It is often associated with elevation of intraocular pressure (IOP) predominantly caused by impaired outflow of aqueous humor [1]. Current medical and surgical managements primarily aim to lower the IOP. The outcome of filtration surgeries, such as trabeculectomy, and glaucoma drainage device (GDD) implantations relies heavily on a functioning bleb to shunt fluid away from the original obstructed aqueous outflow pathway. Poor wound healing, including fibrosis, poses the greatest challenge to post-filtration surgical outcomes, with studies finding long-term success to be 56–73% by 10 years [2,3]. Although bleb failure via postoperative scar formation can be reduced [4,5,6,7] with adjunct anti-scarring agents, such as mitomycin C (MMC) or 5-fluorouracil (5-FU), utilizing these agents can lead to complications, such as bleb leakage, hypotony, and intraocular infection. Therefore, other anti-fibrotic approaches with less deleterious complications have been explored in recent years, including collagen matrix implants [8,9], anti-VEGF agents [10], and the targeting of noncoding RNAs.

Noncoding RNAs, such as microRNAs (miRNA; miR) and long noncoding RNAs (lncRNAs; LINC) are emerging regulators of bleb scarring following filtration surgeries. miRNAs are single-stranded molecules (~19–24 nucleotides long) that negatively regulate gene expression [11] by binding to the 3′ untranslated region of messenger RNAs (mRNAs) to destabilize a transcript [12]. Active in the cytoplasm, miRNAs regulate most cellular processes including differentiation, proliferation, apoptosis, metabolism, and tissue development [13,14]. miRNAs are not only found in cells and tissues but also in biofluids, such as tears [15] and aqueous humor [16]. A single miRNA can have hundreds of effects on downstream mRNAs, and multiple unique miRNAs can modulate one mRNA [17]. These molecules are grouped based on the target interaction: for example, miR-200a and miR-200b, though structurally related, are in separate families due to their differing targets [12].

lncRNAs are 200+ nucleotide-long segments primarily located in the nucleus that modify translational, transcriptional, and epigenetic gene expression [18]. lncRNAs can be subclassified based on the attachment to miRNAs and the direction of transcription [18]. The regulatory mechanisms are diverse and occur at multiple levels, ranging from interactions with mRNAs to affect translation, to the recruitment of histone-modifying enzymes to activate or repress transcription [19]. Historically dismissed as ‘transcriptional noise’, recent studies have shown that lncRNAs have a diverse range of functions and play a regulatory role in numerous ophthalmological diseases, such as glaucoma, corneal neovascularization, cataract, and diabetic retinopathy [18].

Following glaucoma filtering surgery, the process of scar formation within the outflow tract is caused by fibroblast proliferation and extracellular matrix (ECM) accumulation. In the eye, human tenon fibroblasts (HTF) in the ECM are induced by transforming growth factors, such as TGF-β, to differentiate into myofibroblasts that perform secretory and contractile functions [20,21]. Downstream from TGF-β, connective tissue growth factor (CTGF) mediates ECM accumulation and positively feeds back to further promote TGF-β [22]. Rho-associated protein kinase (ROCK) is key to collagen contraction in the trabecular meshwork [23]. Profibrotic cytokines, such as tumor necrosis factor-α (TNF-α), vascular endothelial growth factor (VEGF), and interleukins 1, 6, and 8, are also reported to be upregulated [20,24]. Conversely, protective factors, such as matrix metalloproteinases (MMPs) are downregulated in fibrosis [25]. Identification and investigation of noncoding RNAs are critical to expanding our understanding of interactions among these growth factors and cytokines during bleb scar formation.

miRNAs and lncRNAs are promising therapeutic targets in the prevention of post-glaucoma filtration surgery fibrosis. In this narrative review, we survey potential anti- and pro-fibrotic mechanisms of noncoding RNAs following glaucoma filtration surgery. We propose that noncoding RNAs play a vital role in pathological changes following trabeculectomy or GDD implantation and should be considered for targeted therapeutic interventions.

## 2. Methods

Given the limited research literature that exists on this emerging topic, a narrative review was undertaken. Publications discussing noncoding RNA and post-glaucoma filtration surgeries were searched for using the PubMed, Medline databases. Additionally, a manual search of the reference lists of primary articles was performed. Literature published up to December 2020 was searched. Keywords and MeSH terms related to “microRNA”, “noncoding RNA”, “lncRNA”, “fibrosis”, “human tenon fibroblasts”, “trabeculectomy”, and “glaucoma” were used in the search. These keywords were searched in controlled vocabulary where possible, along with text in the title, abstract, or author-supplied keyword fields. Several exclusion criteria were then applied to the search results, Duplicated publications were removed prior to review, as were any non-English, and/or inaccessible papers.

After initial screening, 38 articles were identified. Articles were evaluated according to their relevance to the review topic and the type of article. From this assessment of abstracts by the authors, 23 papers that focused on the pathophysiology of glaucoma and not post-surgical fibrosis, or were not primary research articles, were excluded. The remaining 15 papers were included in this narrative review. Following manuscript evaluation, papers were sorted into categories based on the type of noncoding RNA, specifically miRNAs and lncRNAs. Within each group of noncoding RNA, manuscripts were further categorized based on anti-fibrotic and pro-fibrotic effects (Table 1). Four of the included papers used microarrays and qRT-PCR to identify up- or down-regulated miRNA profiles and direct further study, with the remainder focusing on miRNAs or lncRNAs based on previous work in other organs or disease processes.

There are several important limitations to the methodology of this narrative review. We discuss a small number of primary research articles with limited scope on the topic of noncoding RNAs following glaucoma filtration surgery. This small body of peer-reviewed primary research literature focusing specifically on glaucoma in the eye makes the comparison of noncoding RNAs in other organs challenging. While each study was appraised individually, objective comparisons between studies are unable to be made given the methodology of this narrative review.

## 3. Results and Discussion

### 3.1. microRNAs

**Figure 1:**
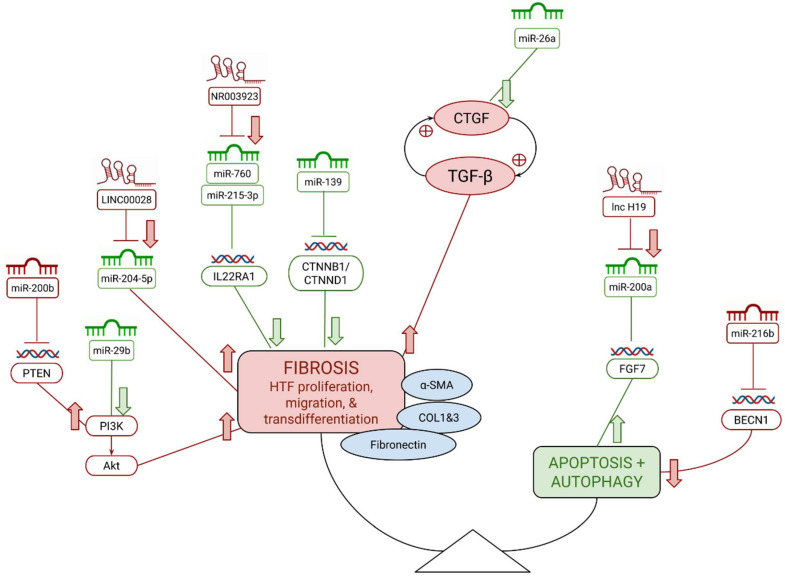
Pathological changes in bleb scarring as a balance between fibrosis and apoptosis, simplified to highlight key roles of discussed miRNAs and lncRNAs. Block arrows reflect final up/down-regulation effects of noncoding RNAs. Red symbolizes a pro-fibrotic role, whereas green reflects anti-scarring. Pictograms are used to visually represent miRNA, lncRNA, and genes.

miR-26a, -29b, -139, -155, and -200a may have anti-fibrotic effects post-trabeculectomy [26,27,28,29,30]. In contrast, miRs-200b and -216b are shown to play pro-fibrotic roles [31,32,33]. Figure 1 summarizes our findings of miRNA contributions to pathological fibrosis in the eye after glaucoma filtration surgery. Individual miRNAs are discussed in more detail below.

### 3.2. Antifibrotic miRNAs

#### 3.2.1. miR-26a

miR-26a is expressed in normal ocular tissue throughout the ciliary body, cornea, lens, and trabecular meshwork [34]. It has been shown to inhibit fibrosis of the lens and the development of cataracts [35,36]. In vitro and tissue profiling studies [26,30] provide strong evidence that miR-26a has a protective role in glaucoma filtration tract fibrosis. miR-26a is differentially expressed in fibrotic bleb tissue isolated from patients after filtration surgery and compared to controls it is downregulated in scar formation [26,30]. Both studies demonstrated a dose-dependent correlation between TGF-β introduction and HTF proliferation via up-regulation of CTGF. Conversely, other studies have found a time-dependent [29] or both dose- and time-dependent relationship [37]. When transfected into HTFs treated with TGF-β, the miR-26a mimics group had anti-scarring effects by inhibiting HTF proliferation and migration. Furthermore, miR-26a also induced apoptosis in aberrant HTFs [30]. These actions appear to be carried out by targeting and interfering with known profibrotic cytokine CTGF.

#### 3.2.2. miR-29b

miR-29b is shown to interact with HTFs in the eye [29,38,39]. Overexpression of miR-29b inhibits fibrosis-related gene expression (PI3K, Sp1, and Col1) in HTFs in vitro [38]. miR-29b is significantly downregulated in TGFβ1-stimulated HTFs. Transfection of HTFs with miR-29b inhibits growth and proliferation, causing effective targeted gene silencing [38]. In vivo experiments in trabeculectomy using a rabbit model demonstrated that the miR-29b treated group achieved consistent and significantly lower IOP compared to preoperative levels and had lower IOP than other treatment groups without miR-29b [39]. Through histologic staining, it was also shown that rabbits treated with miR-29b had fewer fibroblasts and less collagen deposition than rabbits in the conventional therapy group treated with anti-scarring mitomycin but no miR-29b.

miR-29b is upregulated by overexpression of Nrf, a transcription factor activated by oxidative stress that acts to stimulate the production of antioxidants and detoxification proteins. Via the Nrf pathway, miR-29b reverses the profibrotic effects of TGF-β on HTF proliferation [29]. This suppression of activated HTFs by miR-29b is discussed by Bao et al. [26], but the authors reached a discrepant conclusion and identified miR-26a to be responsible for stopping TGF-β stimulation of HTFs. This research is discussed further in Section 3.2.1. This will be an interesting area for future investigation into the interaction of these two miRNAs and which miRNA has a greater role in fibrosis.

#### 3.2.3. miR-139

Bioinformatic studies into the pathogenesis of glaucoma have identified miR-139 as a critical regulatory factor that targets key transcription factors and mRNAs [40]. In TGFβ1-induced fibrosis, miR-139 targets factors in the Wnt/β-catenin (*CTNNB1/CTNND1*) signaling pathway [41]. *CTNND1* and *CTNNB1* genes encode for catenin proteins and when overexpressed, result in increased expression of profibrotic proteins, such as collagen 1 and α-SMA [27]. In a pro-fibrotic state, TGFβ1 may stimulate Smad2/3/4 to bind and suppress miR-139, thereby allowing HTF activation and proliferation.

In HTFs cultured during glaucoma filtration surgery, miR-139 overexpression was found to counteract this TGFβ1-induced HTF proliferation by directly inhibiting *CTNND1* and *CTNNB1* expression [27]. Consistent with this proposed pathway, knockdown of Smad2/3/4 also produces similar effects of *CTNNB1/CTNNB1* suppression and decreased fibrotic proteins. These findings provided the basis for further exploration of targeting miR-139 to reduce the pro-fibrotic state following glaucoma filtration surgery.

#### 3.2.4. miR-200a

miR-200a appears to play a protective role in the pathogenesis of fibrosis, compared to the pro-scarring actions of miR-200b [32,37] (see also Section 3.3.1). Both miR-200a and miR-200b are expressed in epithelial cells [42]. miR-200a regulates the fibroblast growth factor 7 (*FGF7*) gene involved in the *MAPK* pathway for fibrosis [28]. In addition to the role of *MAPK* in fibrosis, inhibition of the *MAPK* can result in the suppression of retinal ganglion cell (RGC) apoptosis, potentially offering another therapeutic target in glaucoma [43]. In a glaucoma mouse model treated with miR-200a mimic, overexpression of miR-200a resulted in *FGF7* downregulation and decreased rates of RGC apoptosis [28]. Silencing miR-200a resulted in a significantly thinner and less dense RGC layer in histological sections of retinal tissue [28]. These pathological changes in retinal tissue and RGC apoptosis could be recovered with upregulated miR-200a and downregulated *FGF7*. The findings suggest both the detrimental role of *FGF7* and the significant protective role of miR-200a in fibrosis. These results are further supported by the work of Zhu et al. [21] on pro-fibrotic lncRNA H19, which exerts its effects by knocking down miR-200a and is discussed further in Section 3.2.1.

### 3.3. Pro-Fibrotic miRNAs

#### 3.3.1. miRNA-200b

miR-200b may promote fibrosis in the glaucoma filtering tract in contrast to miR-200a. Increased miRNA-200b expression in HTFs treated with TGF-β was demonstrated in post-trabeculectomy scarring [37]. Further investigation with this assay showed that miR-200b acts on p27/kip1 and RND3, which are involved in regulating cell proliferation [37]. Studies to explore the mechanism by which miR-200b contributes to fibrosis demonstrated that inhibition of *PTEN*, an inhibitor of the PI3K/Akt pathway, resulted in a corresponding increase in expression of profibrotic proteins P13K, Akt, α-SMA, and fibronectin [32]. These findings suggest that miR-200b acts through different pathways, and it is unclear how many genes are affected by miR-200b and what downstream effects their interactions may have.

#### 3.3.2. miR-200c

miR-200c is another member of the miR-200 family that has been found to mitigate IOP in glaucomatous rats through the regulation of trabecular meshwork cell contraction [44]. Its gene targets include *ZEB1/2*, *FHOD1*, *LPAR1/EDG2*, *ETAR*, and *RHOA* [44]. In our review of the literature, there have been no studies looking into the role of miR-200c in filtration tract scarring in glaucoma patients. This would be an interesting future area of research, given the compelling evidence for miR-200a and miR-200b as players in fibrosis.

#### 3.3.3. miR-216b

The role of miR-216b in regulating HTFs was uncovered while investigating the mechanism of action for an anti-scarring drug hydroxycamptothecin (HCPT) [33]. miR-216b inhibits the beclin1 (*BECN1*) gene, which acts to enhance HTF autophagy and apoptosis [33]. Silencing miR-216b resulted in increased apoptosis-specific proteins in HCPT-treated HTFs. The findings suggest that the miR-216b/Beclin1 axis controls HTF viability, making it an important target for anti-proliferative therapies. There are no current studies demonstrating the relationship between miR-216b and fibrosis following trabeculectomy. Further study is needed to elucidate the contributions of miR-216b to wound healing and scarring.

### 3.4. Long Noncoding RNAs

lncRNAs including H19, NR003923, and 00,028 may have pro-fibrotic effects on the eye [18,21,45]. Figure 1 summarizes our findings of lncRNA contributions to pathological fibrosis in the eye after glaucoma filtration surgery. Individual lncRNAs are discussed in more detail below.

### 3.5. Pro-Fibrotic lncRNAs

#### 3.5.1. H19

H19 was the first discovered lncRNA, consisting of 2300 nucleotides and is located on the 11p15.5 chromosome [46]. Zhu et al. [21] demonstrated that H19 expression can be stimulated by TGF-β in HTFs. Its inhibition resulted in decreased ECM proteins including collagen, fibronectin, and α-SMA. H19 knockdown additionally reduced HTF proliferation. These findings suggested that H19 is a pro-fibrotic lncRNA [21]. Bioinformatic analysis shows that H19 acts by binding and inhibiting miR-200a. Silencing H19 allows miR-200a to suppress β-catenin, resulting in decreased expression of Col1, fibronectin, and α-SMA. The role of this pathway was confirmed with an in vivo rat model that has undergone glaucoma filtration surgery. Levels of H19 and ECM proteins were increased compared to the non-surgical control group, suggesting aberrant expression after glaucoma filtration surgery. The H19/miRNA-200a/β-catenin axis has exciting potential for modulation in regulating fibrosis post-trabeculectomy [21]. These findings are complementary to those of Peng et al. [28] and provide a greater understanding of miR-200a’s anti-scarring role and its interactions with lncRNAs.

#### 3.5.2. NR003923

NR003923 has been identified as upregulated in HTFs and shown to induce pathological processes including TGF-β-induced cell proliferation, migration, and fibrosis [47]. Microarray analysis demonstrated that NR003923 exerts its inhibitory effects on the levels of miR-760 and miR-215-3p [47]. These miRNAs bind to the 3′UTR of the *IL22RA1* gene to inhibit its expression, resulting in downstream effects on HTFs. *IL22RA1* is upregulated in glaucomatous eyes and encodes a receptor for interleukin 22, which has been shown to be protective against fibrosis in other organs [48,49]. When NR003923 suppresses miR-760 and miR-215-3p, there is an upregulation of HTF proliferation, migration, autophagy, and fibrosis [47]. The data from this study indicate that inhibiting NR003923 suppresses TGF-β induced fibrosis, presenting a potential target for improving glaucoma filtration surgery outcomes.

#### 3.5.3. LINC00028

Long intergenic non-protein coding RNA 28 (LINC00028) has recently been discovered to play a pro-fibrotic role in glaucoma and TGF-β induced HTFs [45]. LINC00028 is significantly upregulated in HTFs treated with TGF-β. Knocking down LINC00028 in HTF samples resulted in decreased HTF proliferation, migration, and invasion. miR-204-5p was identified to mediate LINC00028’s pro-fibrotic effects via multiple binding sites in the miR-204-5p sequence. As with NR003923 and its related miRNAs, miRNA-204-5p is protective against fibrosis: miR-204-5p expression is suppressed in TGF-β treated HTFs, but when reintroduced into these tissues, fibrotic elements, such as α-SMA and fibronectin were decreased [45].

## 4. Conclusions

Noncoding RNAs are an exciting area of research in the prevention of outflow tract fibrosis following glaucoma filtration surgery because of their gene regulatory roles in fibroblasts. Further investigation is required to understand their contributions to the pathogenesis of the disease, interactions with other elements, and potential for therapeutic interventions. In the last 15 years, our body of knowledge regarding miRNAs and lncRNAs has grown exponentially. We recognize the vast range of actions that noncoding RNAs exert on target genes, especially in fibrosis. In this review, we highlighted numerous noncoding RNAs involved in fibrosis after glaucoma surgery. Although some of the discussed studies in this review used non-human or non-ocular tissues and thus cannot be fully extrapolated to fibrosis in the eye post-filtration surgery, many of the known miRNAs have preferentially conserved interactions with most human mRNAs and are conserved across animals [50]. Of the studies identified in this narrative review, only four used primary data sets to identify previously unstudied noncoding RNAs in the eye. miRNA microarrays have a number of challenges including high variability in signal-to-noise ratio and the inability to detect novel miRNAs [51,52]. RNA sequencing of miRNAs and lncRNAs allowing for both discovery of new miRNAs and confirmation of known miRNAs could be used in future studies [53]. Further investigation into promising miRNAs associated with fibrosis in other diseases, such as miR-155 [31,54,55] is needed.

Multiple strategies for miRNA-based therapies have been explored. Creating exogenous miRNA mimics may allow us to selectively target gene expression. For miRNAs with deleterious actions, it is possible to use anti-miRNA molecules to knockdown a specific miRNA, and therefore, limit its downstream effects [56]. The potential risk with these approaches includes unintended effects on other genes. Potential limitations to these approaches include the limited impact on target genes due to redundant and/or collateral pathways [11,57]. miRNA-based drugs currently being developed for other diseases are in clinical trials, but none have broken through to common clinical practice to date [58]. Polymeric vectors, viral vectors, and lipid nanoparticles have recently been studied as delivery systems for miRNA mimics or anti-miRNA molecules [59]. 5-FU and MMC remain the standard option for tackling post-surgical fibrosis. Synthetic antisense oligonucleotide ISTH0036 is in preclinical trials as an alternative [60]. Alternative direct targeting of TGF-β with monoclonal antibodies has not yet proven effective [61]. The TGF-β signaling pathway is critical to the maintenance of a range of nonpathological functions, and thus mitigating specific deleterious downstream effects of TGF-β may offer a more targeted approach [62]. There are no anti-scarring miRNA therapies in development for glaucoma filtration surgery, and this will be an emerging space as research continues to grow.

In comparison to the discussed miRNAs, our understanding of lncRNAs is in its infancy. lncRNAs that are strongly implicated in other ocular diseases involving the cornea, lens, and retina include NR033585 [63], HOTAIR [64], and lncRNA-MALAT1 [65]. Research into the role of lncRNAs in the pathogenesis of POAG remains in its initial stages [66,67,68] and lncRNAs have not yet been studied in the context of glaucoma filtration surgery. Further study is needed to elucidate the role these regulators may play in glaucoma surgical outcomes.

**Table 1:** Summary of associated studies for noncoding RNAs discussed.

Noncoding RNA	Studies	Pro/Anti-Fibrotic Role
miR-26a	Wang, Deng, and He, 2018 [30] Bao et al., 2018 [26]	Anti-fibrotic
miR-29b	Li et al., 2012 [38] Ran, Zhu, and Feng, 2015 [29]	Anti-fibrotic
miR-139	Deng et al., 2019 [27]	Anti-fibrotic
miR-200a	Peng et al., 2019 [28]	Anti-fibrotic
miR-200b	Tong et al., 2014 [37] Tong et al., 2019 [32]	Pro-fibrotic
miR-216b	Xu et al., 2014 [33]	Pro-fibrotic
lnc H19	Zhu et al., 2020 [21]	Pro-fibrotic
lnc NR003923	Zhao et al., 2019 [47]	Pro-fibrotic
LINC00028	Sui et al., 2020 [45]	Pro-fibrotic
